# Transcription of Liver X Receptor Is Down-Regulated by 15-Deoxy-Δ^12,14^-Prostaglandin J_2_ through Oxidative Stress in Human Neutrophils

**DOI:** 10.1371/journal.pone.0042195

**Published:** 2012-10-24

**Authors:** Gonzalo Alba, María Edith Reyes, Consuelo Santa-María, Remedios Ramírez, Isabel Geniz, Juan Jiménez, José Martín-Nieto, Elízabeth Pintado, Francisco Sobrino

**Affiliations:** 1 Departamento de Bioquímica Médica y Biología Molecular, Universidad de Sevilla, Sevilla, Spain; 2 Departamento de Bioquímica y Biología Molecular, Universidad de Sevilla, Sevilla, Spain; 3 Distrito Sanitario Sevilla Norte, Servicio Andaluz de Salud, Sevilla, Spain; 4 Departamento de Fisiología, Genética y Microbiología, Universidad de Alicante, Alicante, Spain; Fundação Oswaldo Cruz, Brazil

## Abstract

Liver X receptors (LXRs) are ligand-activated transcription factors of the nuclear receptor superfamily. They play important roles in controlling cholesterol homeostasis and as regulators of inflammatory gene expression and innate immunity, by blunting the induction of classical pro-inflammatory genes. However, opposite data have also been reported on the consequences of LXR activation by oxysterols, resulting in the specific production of potent pro-inflammatory cytokines and reactive oxygen species (ROS). The effect of the inflammatory state on the expression of LXRs has not been studied in human cells, and constitutes the main aim of the present work. Our data show that when human neutrophils are triggered with synthetic ligands, the synthesis of LXRα mRNA became activated together with transcription of the LXR target genes ABCA1, ABCG1 and SREBP1c. An inflammatory mediator, 15-deoxy-Δ^12,14^-prostaglandin J_2_ (15dPGJ_2_), hindered T0901317-promoted induction of LXRα mRNA expression together with transcription of its target genes in both neutrophils and human macrophages. This down-regulatory effect was dependent on the release of reactive oxygen species elicited by 15dPGJ_2_, since it was enhanced by pro-oxidant treatment and reversed by antioxidants, and was also mediated by ERK1/2 activation. Present data also support that the 15dPGJ_2_-induced serine phosphorylation of the LXRα molecule is mediated by ERK1/2. These results allow to postulate that down-regulation of LXR cellular levels by pro-inflammatory stimuli might be involved in the development of different vascular diseases, such as atherosclerosis.

## Introduction

Liver X receptors (LXRs) are oxysterol-activated nuclear receptors active in different human and rodent cells, such as macrophages, adipocytes, hepatocytes, skin fibroblasts and myotubes, which regulate the expression of genes linked to cholesterol metabolism in a tissue-specific manner [1], [2]. This includes the modulation in peripheral blood cells, such as macrophages, of the transcription of a panel of genes encoding proteins involved in reverse cholesterol transport, such as the ATP-binding cassette (ABC) transporters ABCA1 and ABCG1, the lipoproteins ApoE and ApoC, and the lipoprotein-remodelling enzyme PLTP [3], [4]. Systemic activation of LXR thus initiates a series of tissue-specific transcriptional programs aimed at regulating whole-body cholesterol content. For instance, in the intestine LXR controls the reabsorption of cholesterol via activation of ABCG5 and ABCG8 gene expression [5]. Pharmacological activation of LXRs *in vivo* thereby results in increased high-density lipoprotein (HDL) levels, net whole-body cholesterol loss, and reduced atherosclerosis [6], [7].

In addition to their key role in cholesterol homeostasis, LXRs have emerged as important regulators of genes involved in the inflammatory response and innate immunity. Ligand-activated LXR thus blunts the induction of classical pro-inflammatory genes, such as those encoding inducible nitric oxide synthase (NOS2), cyclooxygenase-2 (COX-2), matrix metalloproteinase-9 (MMP-9) and various chemokines in response to stimulation by bacterial lipopolysaccharide (LPS), tumor necrosis factor-α (TNFα) and interleukin-1β (IL-1β), in different mouse cell lines [8], [9]. Other studies have also shown that activation of Toll-like receptors (TLR) 3 and 4 expression by bacterial and viral components results in inhibition of LXR signalling via interferon regulatory factor-3 (IRF-3), a transcription factor, suggesting that LXR functions to modulate crosstalk between inflammatory and metabolic pathways [10], [11] and that the innate immune response is under the control of the LXR pathway [12], [13]. Moreover, LXR ligands have proven effective in the amelioration of inflammation in a number of *in vivo* assays, including animal models of contact dermatitis and atherosclerosis [9], [14], [15]. In addition, activation of LXR attenuates the LPS-induced release of TNFα and prostaglandin E_2_ (PGE_2_) in rat Kupffer cells [16]. However, opposite data have also been reported on the consequences of LXR activation by oxysterols. In this light, the treatment of human peripheral blood monocytes or the differentiated macrophage cell line THP-1 with an LXR ligand, 22(R)-hydroxycholesterol, results in the specific induction of the potent pro-inflammatory cytokine TNFα [17]. Also, LXR activation increased the generation of reactive oxygen species (ROS) by enhancing the expression of NADPH oxidase subunits in primary human macrophages [18]. In the endoplasmic reticulum of human aortic smooth muscle cells the NAD(P)H oxidase subunit Nox-4 mediates 7-ketocholesterol-induced stress [19]. Also in this context, in U937 human promonocytic leukemia cells oxysterols favour the establishment of a pro-oxidant state, and a significant O_2_.^−^ overproduction can be measured in the presence of LXR ligands, such as 7β-hydroxycholesterol or 7-ketocholesterol [20].

15-Deoxy-Δ^12,14^-prostaglandin J_2_ (15dPGJ_2_) is an immunoregulatory lipid metabolite derived from prostaglandin D_2_ (PGD_2_) dehydration *in vivo* which is abundantly produced by mast cells, dendritic cells, and alveolar macrophages [21]. As a ligand for the peroxisome proliferator-activated receptor-γ (PPARγ), 15dPGJ_2_ exerts anti-inflammatory effects that modulate vascular inflammation and atherosclerosis processes [22], [23]. However, this property of 15dPGJ_2_ remains a controversial matter, and caution must be taken in assigning this molecule a solely anti-inflammatory role, given that experimental evidence is also available that 15dPGJ_2_ can induce the synthesis of the type II-secreted pro-inflammatory mediators phospholipase A [24] and COX-2 in vascular smooth muscle [25] and mammary epithelial cells [26]. Moreover, it has been shown that in stimulated human T lymphocytes 15dPGJ_2_ promotes a significant increase in interleukin-8 (IL-8) production through the activation of MAP kinase- and NF-κB-dependent signalling pathways [27], and that 15dPGJ_2_ also induces the expression of IL-8 in human endothelial cells [28]. Furthermore, it has been shown that this prostaglandin, in contrast to reports in macrophages and other cell types, acts as a potent activator of eosinophils by inducing Ca^2+^ mobilization, actin polymerization and CD11b expression [29]. In the same line, data from the present study and a previous report from our laboratory indicate that 15dPGJ_2_ exerts a pro-inflammatory action in human neutrophils, where it elicits the production of high levels of ROS and induction of heme oxygenase 1 (HO-1) expression [30]. These facts are in keeping with previous observations in human neuroblastoma cells [31]. Furthermore, similar data have been reported by our group in human lymphocytes [32] which are also in line with recent observations that in human neutrophils and leukemic Jurkat cells ROS production becomes enhanced in response to 15dPGJ_2_ treatment [33].

Although conflicting data are available on the consequences of LXR activation by oxysterols on the cellular inflammatory state, the opposite mechanism, that is, the potential effect of oxidative stress on LXR gene transcription, has not been analyzed in human circulating neutrophils and constitutes the aim of the present study. In this context, the pivotal role played by neutrophils as a source of ROS and mediators promoting oxidative stress and inflammation, and thereby contributing to the development of atherosclerosis, has been recently unveiled [34]. Also, classical observations had shown that neutrophils, although considered as cells with little protein synthesis capability, are able to synthesize *de novo* a series of cytokines, such as IL-1, IL-8 and interferon-α (IFNα), which are released at sites of inflammation and likely exert a crucial role in host defence mechanisms and against tissue damage [35]. These cells, given their high capacity to synthesize O_2_. ^−^, offer a natural approach to analyze the potential effect of ROS on LXR transcription in human cells, by using 15dPGJ_2_ as a pro-inflammatory molecule. We here report for the first time that human neutrophils when triggered with LXR synthetic ligands display a notable capacity to activate LXRα gene expression, in agreement with other cell types, together with the transcription levels of its target genes ABCA1, ABCG1 and SREBP1c. We describe as well that the presence of 15dPGJ_2_ significantly inhibited the transcription of LXRα and ABC genes elicited by the synthetic LXR ligand, T0901317, and provide data on the intracellular signalling mechanisms involved in regulation of LXRα expression and activity, including oxidative stress and LXRα phosphorylation. Our results thus allow to extend the sphere of influence of LXR to human circulating neutrophils.

## Methods

### Ethics Statement

Peripheral venous blood was drawn from healthy volunteers following standardized protocols approved by the Research Ethics Committee of the Hospital Virgen Macarena, Universidad de Sevilla. The present study meets the requirements for experimentation on human beings, specifically referred to the anonymous blood samples collection from healthy volunteers, whose consent was verbal, and additionally complies with the overall Spanish and European legislation in force, according to the corresponding ethical report emitted by the Universidad de Sevilla. The University Ethics Committee approved the use of a verbal consent protocol with the aim of easing the procedure of sample obtaining and management, as soon as its providers were kept anonymous at all times. Blood withdrawing followed a routinary hospital protocol, and at the moment a simple verbal consent was requested to patients in our hospital. The blood obtained was not solely used for research purposes. Measures taken to document the process included that data handling was strictly kept anonymous and that our group did not have access to patients data unrelated to our study. The investigation was designed and conducted according to the ethical principles for medical research stated in the Declaration of Helsinki.

### Chemicals and Reagents

Phorbol 12-myristate 13-acetate (PMA), phenylarsine oxide, reduced glutathione (GSH), diethylester maleic acid (DEM), 2,2,6,6-tetramethyl-piperidine-1-oxyl (TEMPO), formyl-Met-Leu-Phe (fMLP), diisopropyl fluorophosphate (DFP), IL-1β, IL-8, TNFα, SP600125, and the vitamin E (Vit E) analog 6-hydroxy-2,5,7,8-tetramethylchroman-2-carboxylic acid were purchased from Sigma-Aldrich (Madrid, Spain). SB203580 and PD098059 were products of Calbiochem (San Diego, CA), and RPMI 1640 was obtained from Biomedia (Boussens, France) Fetal calf serum was obtained from BioWhittaker (Basel, Switzerland). Polyvinylidene difluoride (PVDF) membranes were from Pall (Madrid, Spain), and Dextran T-500 and lymphocyte separation medium (Ficoll-Paque) were obtained from GE Healthcare (Barcelona, Spain). T0901317 and prostaglandins PGA_1_, PGA_2_, PGD_2_ and PGE_2_ were products of Cayman Chemical (Ann Arbor, MI). 15dPGJ_2_ was obtained from Biomol (Plymouth Meeting, PA). Rabbit polyclonal antibodies against phosphorylated (Thr202/Tyr204) extracellular signal-regulated kinases 1 and 2 (ERK1/2) and total (unphosphorylated plus phosphorylated) ERK1/2 were obtained from New England Biolabs (Beverly, MA). Mouse monoclonal anti-human LXRα was obtained from Perseus Proteomics (Tokyo, Japan). Rabbit polyclonal anti-phosphoserine and mouse monoclonal anti-GAPDH were purchased from Chemicon International (Madrid, Spain). Horseradish peroxidase (HRP)-conjugated goat anti-rabbit and anti-mouse IgGs were from Promega (Madison, WI).

### Isolation and Culture of Human Neutrophils

Human peripheral blood neutrophils were isolated as indicated [36], from fresh heparinized blood of healthy human donors after informed consent, and further purified by Dextran T-500 sedimentation, followed by Ficoll-Paque gradient centrifugation and hypotonic lysis of residual erythrocytes. Neutrophils were washed twice in Hank’s balanced salt solution and then suspended at a density of 10^7^ cells/ml in Krebs Ringer Hepes buffer or RPMI 1640 medium supplemented with 10% fetal calf serum plus gentamicin, penicillin and streptomycin at 50 mg/ml each. Before all stimulations, neutrophil suspensions were preincubated at room temperature with 1 mM DFP (to minimize proteolysis) for 5 min [37].

### Isolation and Culture of Human Macrophages

Human monocyte-derived macrophages were obtained from buffy coat preparations by Ficoll density gradient centrifugation, followed by adhesion-mediated purification on tissue culture or gelatin-coated plastic. The monocytes differentiate into macrophages in vitro by culturing in medium containing autologous human fibrin-depleted plasma [38].

### RT-PCR Analyses of mRNA Levels

For RT-PCR analysis, 2 µg of total RNA was reverse-transcribed into cDNA as described [32]. Real time PCR was carried out in an ABI Prism 7300 Sequence Detection System (Applied Biosystems, Foster City, CA) using the specific thermocycler conditions recommended by the manufacturer. PCR reactions were performed in triplicate and contained 2 µl of cDNA and SYBR Green PCR Master Mix (Applied Biosystems) in a total volume of 25 µl, and using the following primers: hLXRα: forward, 5′-AAGCCCTGCATGCCTACGT-3′, reverse, 5′-TGCAGACGCAGTGCAAACA-3′; hLXRβ: forward, 5′-TCGTGGACTTCGCTAAGCAA-3′, reverse, 5′-GCAGCATGATCTCGATAGTGGA-3′; hABCA1: forward, 5′-CCCTGTGGAATGTACCTATGTG-3′, reverse, 5′-GAGGTGTCCCAAAGATGCAA-3′; hABCG1: forward, 5′-CAGTCGCTCCTTAGCACCA-3′, reverse, 5′-TCCATGCTCGGACTCTCTG-3′; hSREBP1c: forward, 5′-TCAGCGAGGCGGCTTTGGAGCAG-3′, reverse, 5′-CATGTCTTCGATGTCGGTCAG-3′; and β-actin: forward, 5′-CCAGCTCACCATGGATGATG-3′, reverse, 5′-ATGCCGGAGCCGTTGTC-3′. Relative levels of transcripts above were quantified by the comparative threshold cycle (Ct) method as described in the ABI Prism 7300 User Bulletin 2 [30], and normalized to β-actin mRNA levels.

### Western Blotting Analysis of LXRα, Phosphorylated ERK1/2 and Phosphoserine Levels

Cells were rinsed once with ice-cold PBS, resuspended in a lysis solution containing 50 mM Tris-HCl (pH 7.4), 10 mM EDTA, 50 mM NaF, 10% glycerol, 1% Triton X-100, 10 µg/ml leupeptin, 10 µg/ml aprotinin and 1 mM phenylmethylsulfonyl fluoride (PMSF), and kept on ice for 30 min. Then the cells were disrupted by sonication on ice and, after centrifugation at 12,000×g for 5 min at 4°C, protein concentration in the supernatant was determined by the Bradford method [39], using bovine serum albumin (BSA) as a standard. Proteins [40] were boiled in Laemmli loading buffer, resolved by SDS-PAGE (10% polyacrylamide) and transferred to PVDF membranes as previously described [41] The blots were probed without need of prior blocking [42] with mouse monoclonal anti-LXRα or with rabbit polyclonal anti-phospho ERK1/2 or anti-phosphoserine antibodies at a 1∶1,000 dilution in PBS plus 0.5% BSA and 0.02% Tween-20. Thereafter, HRP-conjugated antibodies to mouse or rabbit IgG were used at a 1∶5,000 dilution in PBS plus 0.5% casein followed by enhanced chemiluminescence [36]. To verify even protein loading, the blots were subsequently stripped and reprobed with mouse monoclonal antibodies against GAPDH, or rabbit polyclonal antibodies against total ERK1/2 at a 1∶1,000 dilution. Band intensities were measured by scanning densitometry analysis using the Scion Image software (Frederick, MD).

### Immunoprecipitation Analysis of Serine-phosphorylated Proteins

Stimulated cells were pelleted and lysed in 75 µl of ice-cold lysis buffer B, containing 50 mM Tris-HCl (pH 7.4), 1% Triton X-100, 300 mM NaCl, 100 µM phenylarsine oxide, 10 µg/ml leupeptin, 10 µg/ml aprotinin, 1 mM PMSF, 1 mM Na_3_VO_4_ and 5 mM EDTA. The lysates were centrifuged at 12,000×g for 5 min. For immunoprecipitation, incubation with 0.5 µg of anti-LXRα specific antibodies was carried out in a volume of 70 µl with rotation for 2 h at 4°C, followed by addition of 40 µl of a 50% slurry of protein A-Sepharose beads in buffer B and further incubation for 2 h at 4°C. The immune complexes were washed five times with ice-cold lysis buffer B, and the proteins were extracted by boiling the pellet in Laemmli sample buffer. After resolution by SDS-PAGE (10% polyacrylamide), the proteins were electrotransferred to PVDF membranes as previously described [41], and subjected to immunoblotting analysis with antibodies to phosphoserine or LXRα as indicated above.

### ELISA Quantitation of IL-8 Release

Cell culture supernatants were collected after treatments, and the levels of secreted IL-8 were quantified using the Human IL-8 ELISA Kit (Raybiotech, Norcross, GA). Plates were read on a Wallac 1420 Victor^2^ spectrofluorometer (Perkin Elmer, Madrid, Spain).

### Measurement of ROS using Hydroethidine

Neutrophils were treated as described above in the presence of 2 µM hydroethidine. This reagent is intracellularly oxidized by oxygen radicals to yield ethidium bromide, which tightly binds to DNA and emits a strong red fluorescence [43] whose intensity was measured using excitation/emission wavelengths of 550 nm/615 nm in the Wallac 1420 Victor^2^ apparatus (Perkin–Elmer).

### Chemotaxis Assay

Migration of neutrophils was evaluated in Transwell migration chambers (6.5 mm diameter, 5 µm pore size; Costar plates type 3421). Chemoattractants were deposited in the lower compartment in a final volume of 0.6 ml of RPMI 1640, and the plates were prewarmed at 37°C. Then 0.1 ml of medium containing 10^6^ neutrophils was deposited on each detachable insert, which was placed over the chemoattractant solution. Loaded chambers were incubated for 2 h at 37°C in a humidified atmosphere of 5% CO_2_ in air. In separate wells, neutrophils were added to the lower compartment and used as controls representing 100% migration. At the end of the incubation period, the cells that had migrated into the bottom chambers were collected and centrifuged. After staining with FITC-conjugated monoclonal antibodies to CD16 (Immunotech, Marseille, France), the cells were fixed with 1% paraformaldehyde and finally counted on a flow cytometer. The results are presented as the mean ± SEM of three separate experiments, and are expressed as the percentage of total neutrophils initially added to each chamber.

### Cholesterol Efflux

Cholesterol efflux assays were performed as described [44], with modifications. Cells were washed and incubated for 5 h in RPMI 1640 supplemented with 0.2% BSA with [3H]cholesterol (2.0 µCi/ml). To equilibrate cholesterol pools, cells were washed twice with PBS and incubated for 15 hr in RPMI containing 0.2% BSA plus the indicated ligands (1 µM T0901317 or 10 µM 15dPGJ_2_) plus or apoAI (15 µg/ml), but lacking radiolabeled cholesterol. An aliquot of the medium was removed and centrifuged at 14,000×g for 2 min, and the radioactivity was determined by liquid scintillation counting. Total cell-associated radioactivity was determined by dissolving the cells in isopropanol. Each assay was performed in triplicate.

### Detection of Cellular Apoptosis

Apoptosis, measured as DNA fragmentation, was tested using the Cell Death Detection ELISA^plus^ kit from Roche Applied Science (Barcelona, Spain), according to the manufacturer’s instructions. No evidence of apoptotic death was found in our cells, even after 20 h of incubation in our experimental conditions. Neutrophils treated with 100 nM staurosporine were used as positive control.

### Statistical Analyses

mRNA levels quantitated by real time PCR are expressed as fold induction relative to untreated cells (mean ± SEM from a minimum of 3 independent experiments performed with similar results). Protein levels quantitated from Western blots are expressed in arbitrary units. The results were statistically analyzed using the Statgraphics Plus 5.0 software (Manugistic Inc., Rockville, MD) by means of ANOVA and the Student’s paired *t*-test.

## Results

### 15dPGJ_2_ Inhibits the Expression of LXRα and its Regulated Genes in Human Neutrophils

**Figure 1:**
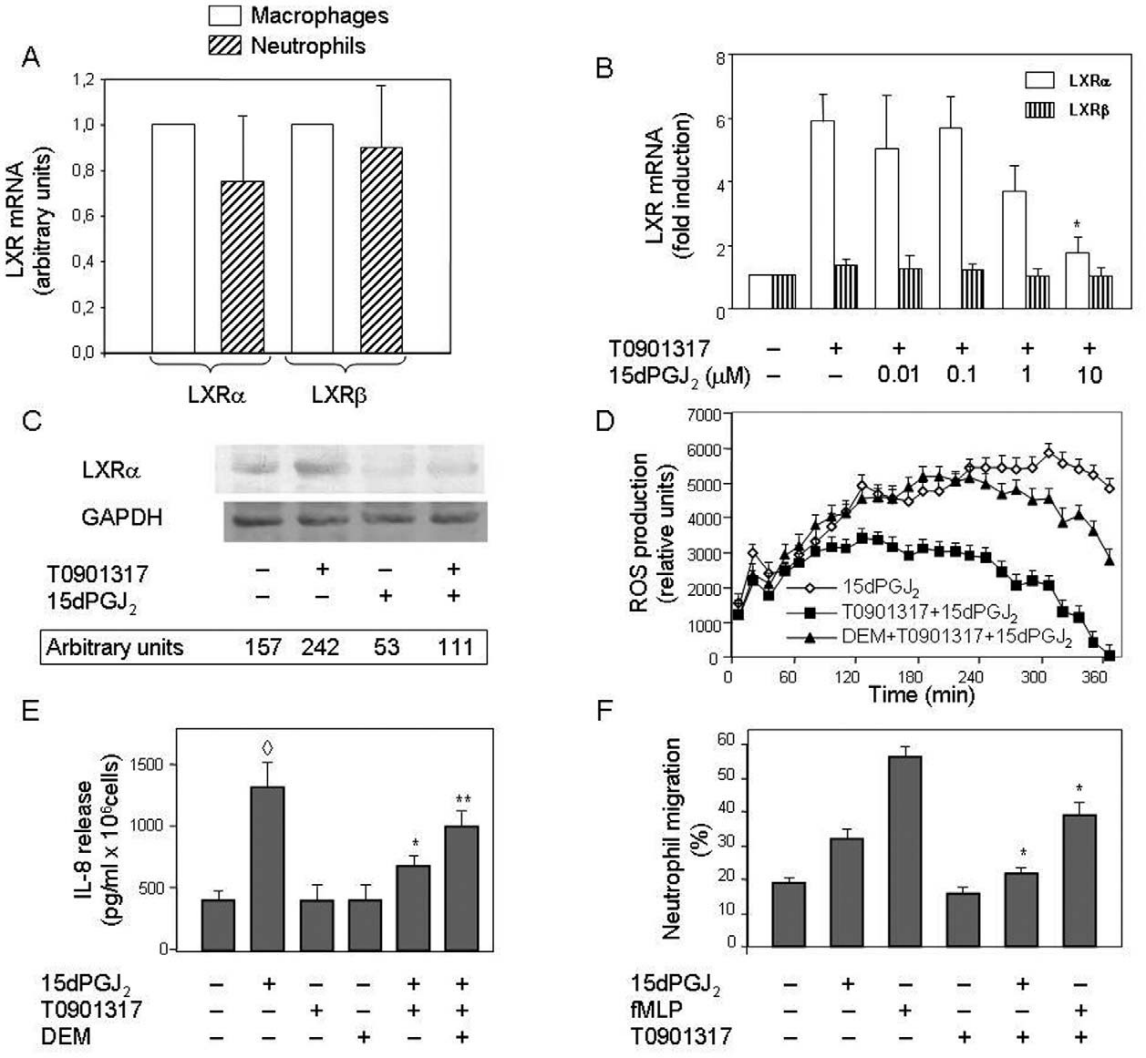
15dPGJ_2_ prevents ligand-induced LXRα mRNA expression in a dose-dependent manner. Neutrophils or macrophages were left untreated (A), or else neutrophils were preincubated at 37°C for 1 h with or without 15dPGJ_2_ at the indicated doses (B) or at a 10 µM concentration (C), and then they were treated or not with T0901317 for 18 h (B and C). Then LXRα and LXRβ mRNA levels were measured by real time RT-PCR (A and B), or LXRα protein levels were analyzed by Western blotting (C). Statistical data from real time RT-PCR experiments were normalized to LXRα or LXRβ levels found in human macrophages (A) and corrected for differences in β-actin mRNA levels (as endogenous gene) and expressed as fold induction (A and B). GAPDH bands in Western blots are shown for the sake of protein loading controls (C). In a different set of experiments, neutrophils were pretreated or not with 10 µM DEM for 30 min (D and E) and, after preincubation in the absence or presence of 1 µM T0901317 for 4 h (D and E), 10 µM 15dPGJ_2_ was added and ROS levels were monitored by luminescence measurement for the next 6 h (D), or released IL-8 levels were quantitated by ELISA after an incubation period of 4 h (E). Neutrophil migration was quantitated in cells preincubated with or without 1 µM T0901317 for 4 h and then transferred to Transwell chambers and treated with 10 µM 15dPGJ_2_ or 0.1 µM fMLP for 1 h. Results are expressed as the percentage of cells that migrated from the upper to the lower compartment (F). Each panel is representative of a set of three experiments yielding similar results. Values are plotted as the mean ± SEM (n = 3) (A, B, D, E and F). * P<0.01 for T0901317-stimulated, 15dPGJ_2_ (B, F) or fMLP (F)-treated versus -untreated. ^◊^ P<0.01 for 15dPGJ_2_-treated versus untreated (E). ** P<0.01 for 15dPGJ_2_ and T0901317-stimulated, DEM-treated versus -untreated (E).

In preliminary experiments, we attempted to transfect human circulating neutrophils with plasmids containing cloned LXR genes or a short interfering RNA (siRNA) specific for the human LXRα. Yet, these experiments were unsuccessful, likely due to the intrisic difficulty posed by these cells to become transfected with DNA. Initial experiments were designed to analyze whether each of the two LXR isoforms, α and β, was present at basal levels in human neutrophils. A relative quantification of LXRα and LXRβ mRNAs in untreated human neutrophils and macrophages by means of real time RT-PCR revealed that these two cell types expressed comparable basal levels of both LXR genes (Figure 1A). However, we found that only the LXRα mRNA was induced in human neutrophils by a synthetic ligand, T0901317, whereas the LXRβ mRNA was constitutively expressed by these cells and its levels remained unchanged (Figure 1B). As shown, the LXRα mRNA levels became up-regulated between 3 and 6 times after treatment for 18 h with T0901317, as measured in human neutrophils by real-time RT-PCR (Figure 1B). Next, experiments were designed to analyze the potential role on this process of 15dPGJ_2_, a ligand of PPARγ with capacity to modify the intracellular redox status, although with conflicting results reported on whether it enhances or ameliorates the inflammatory response. Interestingly, although 15dPGJ_2_ by itself did not exert any positive or negative effect on LXRα mRNA expression (data not shown), it was able to drastically inhibit the stimulation of this process exerted by T0901317 in human neutrophils (Figure 1B), Indeed, 15dPGJ_2_ effectively counteracted T0901317-activated LXRα expression, although only at 10 µM its inhibitory effect was consistently observed and statistically significant, by ca. 75% (Figure 1B). Furthermore, the inhibition of LXRα mRNA expression correlated with decreased levels of LXRα protein in 15dPGJ_2_-treated neutrophils (Figure 1C). A decrease of basal LXRα protein was also seem in the presence of 15dPGJ_2_ alone (Figure 1C), although this small effect was not consistently observed.

We then set to analyze the effect of 15dPGJ_2_ on the intracellular redox status in human neutrophils. To this end, we measured the short-term ROS production by neutrophils incubated with 15dPGJ_2_. It was found that 15dPGJ_2_ was able by itself to induce within minutes the production of ROS by these cells (Figure 1D). Moreover, the LXR synthetic ligand, T0901317, did not induce by itself ROS production (data not shown), although it reduced ROS generation elicited by 15dPGJ_2_ (Figure 1D). These facts suggested that T0901317 did modify the intracellular redox status in human neutrophils. It was noteworthy that under pro-oxidant conditions, i.e. cells treated with diethylester maleic acid (DEM), an oxidant that depletes glutathione in the cell, the effect of T0901317 on ROS generation was reversed (Figure 1D). These data demonstrated that oxidative stress could change an LXR-mediated cell function in human neutrophils. Neither T0901317 nor DEM affected significantly ROS production by themselves (data not shown). In order to further assess the pro-inflammatory properties of 15dPGJ_2_ and anti-inflammatory effects of LXR activation, we studied its effects on the release of IL-8, the main interleukin produced by neutrophils under pro-oxidant conditions. Figure 1E shows that 15dPGJ_2_ strongly induced a significant IL-8 release to the medium by human neutrophils, and that this effect was hindered by subsequent T0901317 treatment. Thus, for both parameters measured, i.e. ROS and IL-8 levels, 15dPGJ_2_ clearly exhibited pro-inflammatory properties in human neutrophils, which were significantly altered by T0901317 treatment. It should be noted that the effect of T0901317 on ROS and IL-8 levels was only observed after a previous treatment for at least 4 h with the ligand. Furthermore, T0901317 treatment for this period also hindered neutrophil migration activity induced by 15dPGJ_2_ or by the chemoattractant formyl-Met-Leu-Phe (fMLP) (Figure 1F), these data demonstrated that LXRα plays a role in regulation of inflammation. Also, we studied its role in cholesterol homeostasis. With this purpose, we analysed the efflux of cholesterol in human neutrophils in the presence and absence of T0901317 and/or 15dPGJ_2_. However, this ligand was unable to alter cholesterol levels (data not shown).

**Figure 2:**
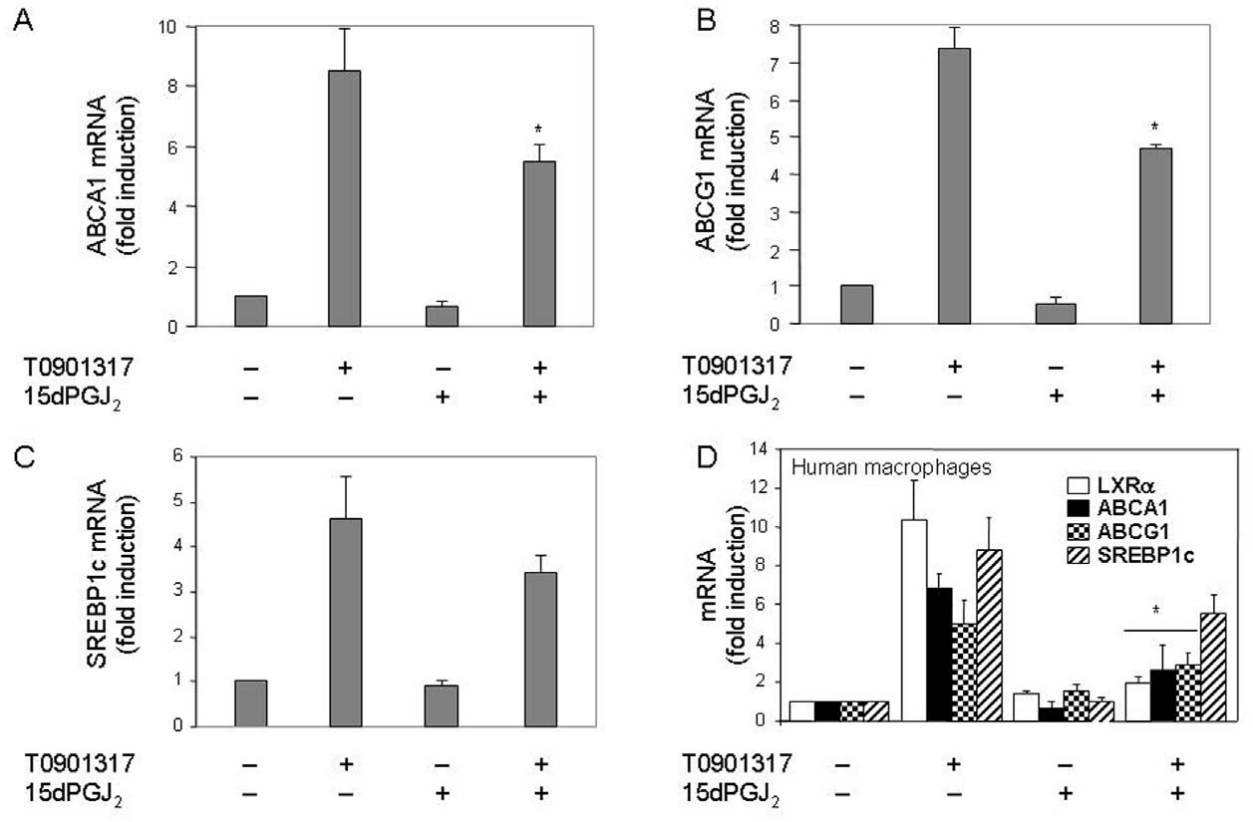
15dPGJ_2_ prevents T0901317-induced ABCA1, ABCG1 and SREBP1c mRNA expression in human neutrophils and macrophages. Neutrophils (A-C) or macrophages (D) were preincubated at 37°C with or without 10 µM 15dPGJ_2_ for 1 h, and thereafter they were stimulated or not with 1 µM T0901317 for 18 h. The levels of mRNA from the indicated genes were analyzed by real time RT-PCR. Statistical data (mean ± SEM, n = 3) were corrected for differences in β-actin mRNA levels and expressed as fold induction. * P<0.01 for T0901317-stimulated, 15dPGJ_2_-treated versus -untreated.

To investigate whether the inhibitory effect of 15dPGJ_2_ was specific for the LXRα in human neutrophils, we examined its biological activity by measuring the mRNA levels of a series of genes activated by this transcription factor, such as those encoding the cholesterol efflux transporters ABCA1 and ABCG1. It was found that the T0901317-activated transcription of these two genes was partially prevented by 15dPGJ_2_ pretreatment (Figure 2, A and B), with an observed inhibition of ca. 40% for each ABCA1 and ABCG1 transcript levels with respect to those measured with T0901317 alone. SREBP1c was another LXR-dependent gene whose transcription, as shown in Figure 2C, was as well hindered by 15dPGJ_2_ in neutrophils stimulated with T0901317, although, in this case such negative effect was not statistically significant. From these experiments it was concluded that transcription of the LXRα and its target genes induced by LXR ligands was inhibited by the simultaneous presence of 15dPGJ_2_ in human neutrophils. Since the majority of previous studies on LXR gene transcription had been performed on macrophages, we set to verify whether the inhibitory effect of 15dPGJ_2_ also took place in this cell type. As illustrated in Figure 2D, 15dPGJ_2_ inhibited as well LXRα, ABCA1, ABCG1 and SREBP1c mRNA transcription in cultured human macrophages treated with T0901317, although, as it was the case for neutrophils, the 15dPGJ_2_ inhibitory effect on SREBP1c was not statistically significant.

### Other Pro-inflammatory Molecules also Inhibit LXRα mRNA Expression in Human Neutrophils

**Figure 3:**
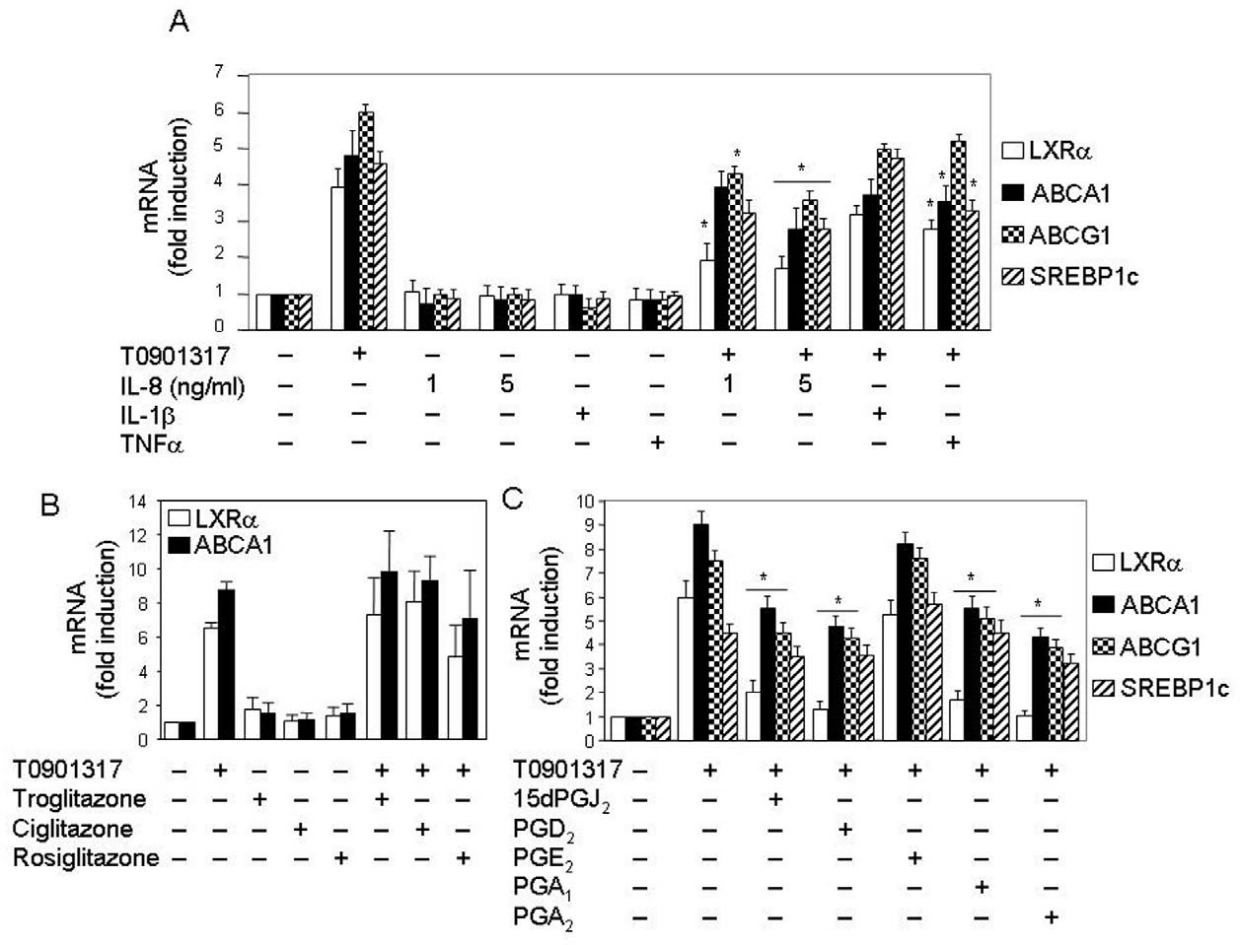
Pro-inflammatory cytokines and prostaglandins, but not PPAR agonist, inhibit induction of mRNA expression of LXRα target genes in human neutrophils. Neutrophils were preincubated at 37°C for 1 h with or without 330 ng/ml IL-1β, 200 ng/ml TNFα or IL-8 at the indicated doses (A), 1 µM troglitazone, 1 µM ciglitazone or 1 µM rosiglitazone (B),or else with 10 µM 15dPGJ_2_, 10 µM PGD_2_, 10 µM PGE_2_, 15 µM PGA_1_ or 25 µM PGA_2_ (C). Then, after incubation in the absence or presence of 1 µM T0901317 for 18 h, the levels of LXRα, ABCA1, ABCG1 and SREBP1c mRNAs were analyzed by real time RT-PCR. Statistical data (mean ± SEM, n = 3) were corrected for differences in β-actin mRNA levels and expressed as fold induction. * P<0.01 for treated with pro-inflammatory cytokines (A) or prostaglandins (C) plus T0901317-stimulated versus untreated, T0901317-stimulated only.

In order to investigate whether the inhibitory effect of 15dPGJ_2_ was attributable to its pro-inflammatory properties, we studied the effect of well known pro-inflammatory cytokines, namely IL-8, IL-1β and TNFα, on LXRα mRNA levels as well as on the expression of LXRα target genes, ABCA1, ABCG1 and SREBP1c. Figure 3A shows that these cytokines not only inhibited the ligand-induced LXRα mRNA expression, but also that of LXRα target genes, ABCA1, ABCG1 and SREBP1c. Yet, the negative effect of IL-1β on the four genes, although observable, was not statistically significant, as it was the case for the TNFα effect on ABCA1 induction (Figure 3A). Interestingly, IL-8 inhibited LXRα mRNA expression at a dose similar to IL-8 levels released upon 15dPGJ_2_ treatment by human neutrophils.

15dPGJ_2_ is a known PPARγ natural ligand. Some authors have described that LXR and PPARγ ligands act in a cooperative manner to induce LXRα expression in human macrophages [1]. Thus, we decided to investigate whether the inhibitory effect of 15dPGJ_2_ was dependent on PPARγ activation. To address this question, we treated neutrophils with three synthetic PPARγ agonists, troglitazone, ciglitazone and rosiglitazone, at a 1 µM concentration. As shown in Fig. 3B, these compounds did not significantly affect either the basal levels of LXRα mRNA expression or its T0901317-induced transcription in human neutrophils. Similar negative results were obtained for ABCA1 expression (Fig. 3B). These observations were suggestive that 15dPGJ_2_ inhibits LXRα expression in a PPARγ-independent manner.

Given that the effects of cyclopentenone prostaglandins, like 15dPGJ_2_ and A-series prostaglandins, are usually mediated by the reactive α,β-unsaturated carbonyl group in their cyclopentenone ring [45], subsequent experiments were addressed to study the effect on LXRα expression of prostaglandins A, which do not bind to any member of the PPAR family of nuclear receptors. Figure 3C illustrates that T0901317-promoted LXRα mRNA synthesis was strongly inhibited by both PGA_1_ and PGA_2_ in human neutrophils. Moreover, PGD_2_, the precursor of 15dPGJ_2_ [31], also prevented LXRα gene expression, whereas PGE_2_ had no significant effect on its induction by T0901317. Similar inhibitory effects were detected for these prostaglandins on the three LXRα target genes analyzed (Figure 3C), although their negative effect on SREBP1c expression was (as in Figure 2D) not statistically significant.

### Inhibition of LXRα Transcription by 15dPGJ_2_ is Exerted through ROS

**Figure 4:**
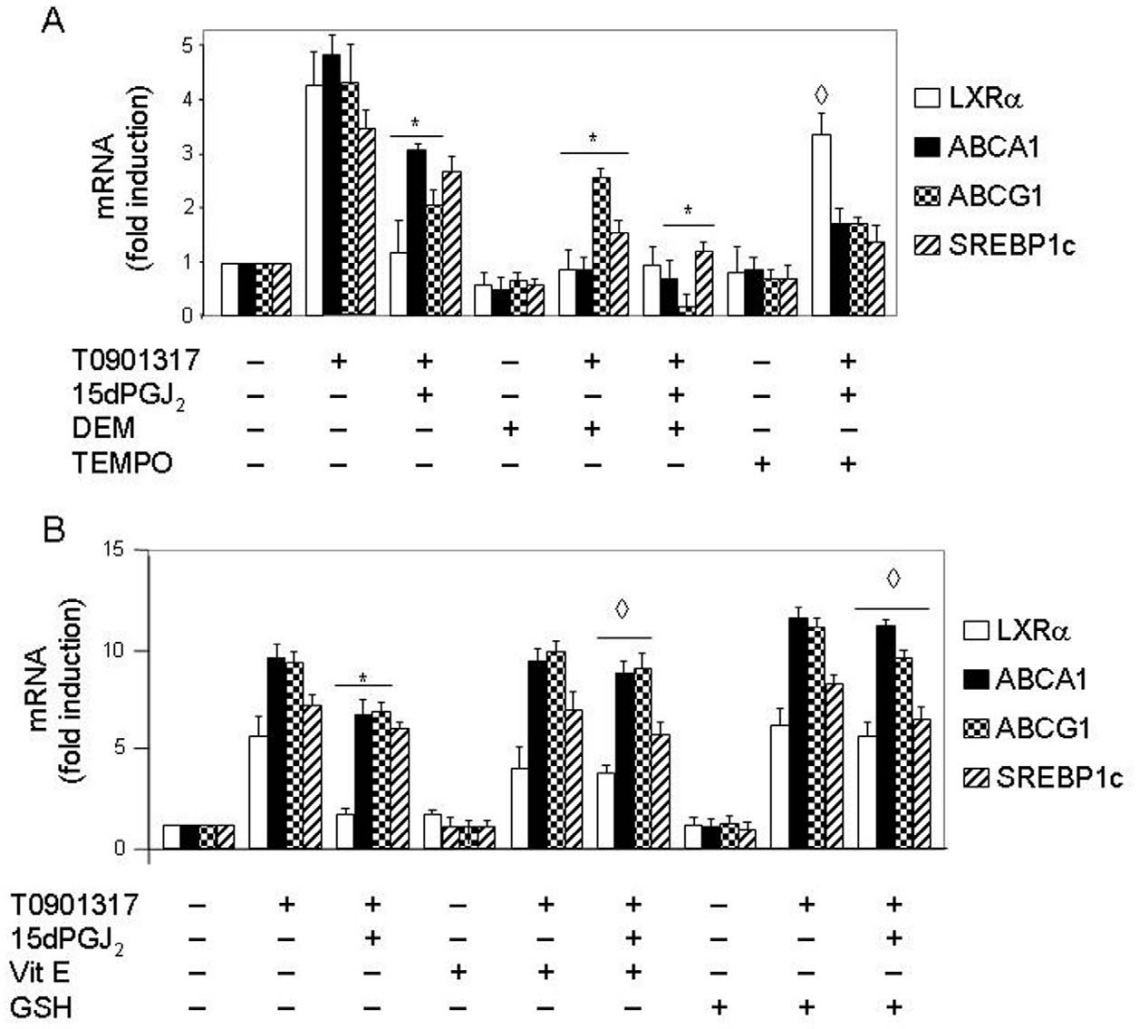
15dPGJ_2_ inhibition of LXR target genes mRNA expression is mediated by oxidative stress in human neutrophils. Neutrophils were preincubated at 37°C with or without 10 µM DEM, 100 µM TEMPO (A), 100 µM vitamin E analog (Vit E) or 1 mM GSH (B) for 1 h. Then the cells were treated or not with 10 µM 15dPGJ_2_ for 1 h, and thereafter stimulated or not with 1 µM T0901317 for 18 h. Finally, the levels of LXRα target genes mRNAs were analyzed by real time RT-PCR. Statistical data (mean ± SEM, n = 3) were corrected for differences in β-actin mRNA levels and expressed as fold induction. * P<0.01 for T0901317–stimulated, 15dPGJ_2_ (A and B) or DEM (A)-treated versus -untreated. ^◊^ P<0.01 for 15dPGJ_2_ and T0901317-stimulated, TEMPO (A), Vit E or GSH (B)-treated versus -untreated.

Our group has shown that some effects of 15dPGJ_2_ are exerted through its intrinsic ability to elicit ROS production by human lymphocytes and neutrophils [30], [31]. We thus tested whether the negative effect of 15dPGJ_2_ on LXRα mRNA expression was affected by oxidative stress. With this purpose, neutrophils were preincubated with either the pro-oxidant agent, DEM, or the reducing molecules 2,2,6,6-tetramethyl-piperidine-1-oxyl (TEMPO) (a ROS scavenger), 6-hydroxy-2,5,7,8-tetramethylchroman-2-carboxylic acid (a soluble Vit E analog), or GSH, prior to addition of 15dPGJ_2_ to the medium, and then LXRα mRNA synthesis was activated with T0901317. Figure 4A illustrates that DEM significantly inhibited by itself T0901317-dependent mRNA expression of the LXRα and its target genes (i.e. ABCA1, ABCG1 and SREBP1c) (P<0.01), and that it was also able to enhance the negative effect of 15dPGJ_2_ on the mRNA levels of LXRα target genes, but not on LXRα expression, possibly due to the strong inhibition exerted by 15dPGJ_2_ by itself (Figure 4A). Even, DEM alone was able to decrease LXRα expression below basal levels, although not significantly. In contrast, reducing molecules such as TEMPO (Figure 4A) and the Vit E analog (Figure 4B) significantly counteracted the negative effect of 15dPGJ_2_ on LXRα mRNA expression with a statistical significant of P<0.01, and GSH totally abolished such effect with a statistical significant of P<0.01 (Figure 4B). Interestingly, the counterbalancing effect of reducing molecules was also significant on the other three LXRα target genes studied, with a statistical significant of P<0.01, except for TEMPO, which was not able to reverse the negative effect of 15dPGJ_2_ on the expression of these genes (Figure 4A). In control experiments, it was found that neither the Vit E analog (Figure 4B) nor TEMPO or GSH (data not shown) alone had any effect on T0901317 stimulatory action. These data strongly suggested that intracellular ROS are involved in the inhibition of LXRα mRNA synthesis exerted by 15dPGJ_2_ in human neutrophils treated with T0901317.

### LXRα Expression is ERK1/2-dependent in Human Neutrophils: Effect of 15dPGJ_2_

**Figure 5:**
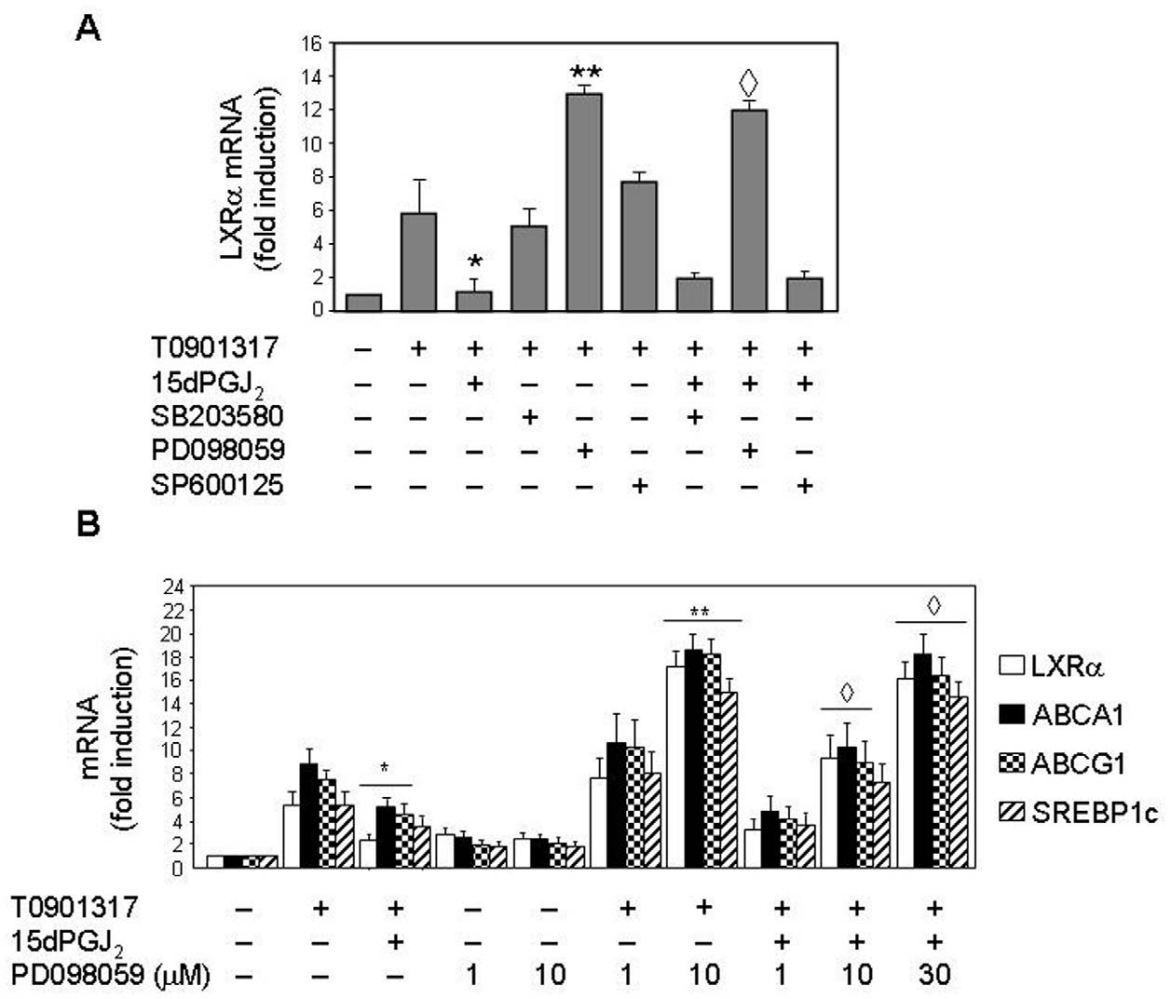
15dPGJ_2_ inhibition of LXR target genes mRNA expression is mediated by ERK1/2 in human neutrophils. Neutrophils were pretreated at 37°C with or without 10 µM SB203580 or 1 µM SP600125, or with PD098059 at 40 µM (A) or at the indicated doses (B) for 1 h. Then the cells were incubated in the absence or presence of 10 µM 15dPGJ_2_ for 1 h, and further stimulated or not with 1 µM T0901317 for 18 h. Finally, the levels of LXR target genes mRNA were analyzed by real time RT-PCR. Statistical data (mean ± SEM, n = 3) were corrected for differences in β-actin mRNA levels and are expressed as fold induction. * P<0.01 for T0901317-stimulated, 15dPGJ_2_-treated versus -untreated. ** P<0.01 for T0901317-stimulated, PD098059-treated versus -untreated. ^◊^ P<0.01 for 15dPGJ_2_ plus T0901317-stimulated, PD098059-treated versus -untreated.

Recently, Tontonoz’s and Garabedian’s groups have established that the LXRα molecule is phosphorylated at a single site, Ser-198, in its hinge region [46], [47]. This serine residue is a target site for mitogen-activated protein kinase (MAPK) phosphorylation. On this basis, we analyzed in subsequent experiments the potential involvement of MAPK pathways in LXR mRNA expression and its down-regulation promoted by 15dPGJ_2_ in human neutrophils. With this aim, neutrophils were incubated with different kinase inhibitors, such as SB203580 (a p38 MAPK inhibitor), PD098059 (an inhibitor of MEK1/2, the upstream activator of ERK1/2), and SP600125 (an inhibitor of c-Jun N-terminal kinases 1 and 2, JNK1/2), prior to addition of 15dPGJ_2_ to the medium. As shown in Figure 5A, neither SB203580 nor SP600125 had any significant effect on T0901317-promoted LXRα transcription, nor did they alter the negative effect of 15dPGJ_2_ on this process. This suggested that the MAPKs p38 and JNK1/2 are not involved in LXRα transcription. However, in neutrophils preincubated with PD098059, the MEK1/2 inhibitor, LXRα target genes mRNA synthesis induced by T0901317 was dramatically enhanced in a PD098059 (Figures 5, A and B). Even, this inhibitor was able by itself to (slightly) induce LXRα target genes mRNA expression above basal levels (Figure 5B), and to potently abrogate the 15dPGJ_2_ inhibitory effect on this process (Figures 5, A and B). These data were indicative that ERK1/2 activation down-regulates LXRα target genes mRNA transcription induced by T0901317, and that the inhibitory effect of 15dPGJ_2_ could be mediated by ERK1/2 activation.

### 15dPGJ_2_ Elicits Redox-dependent Phosphorylation of ERK1/2 in Human Neutrophils

**Figure 6:**
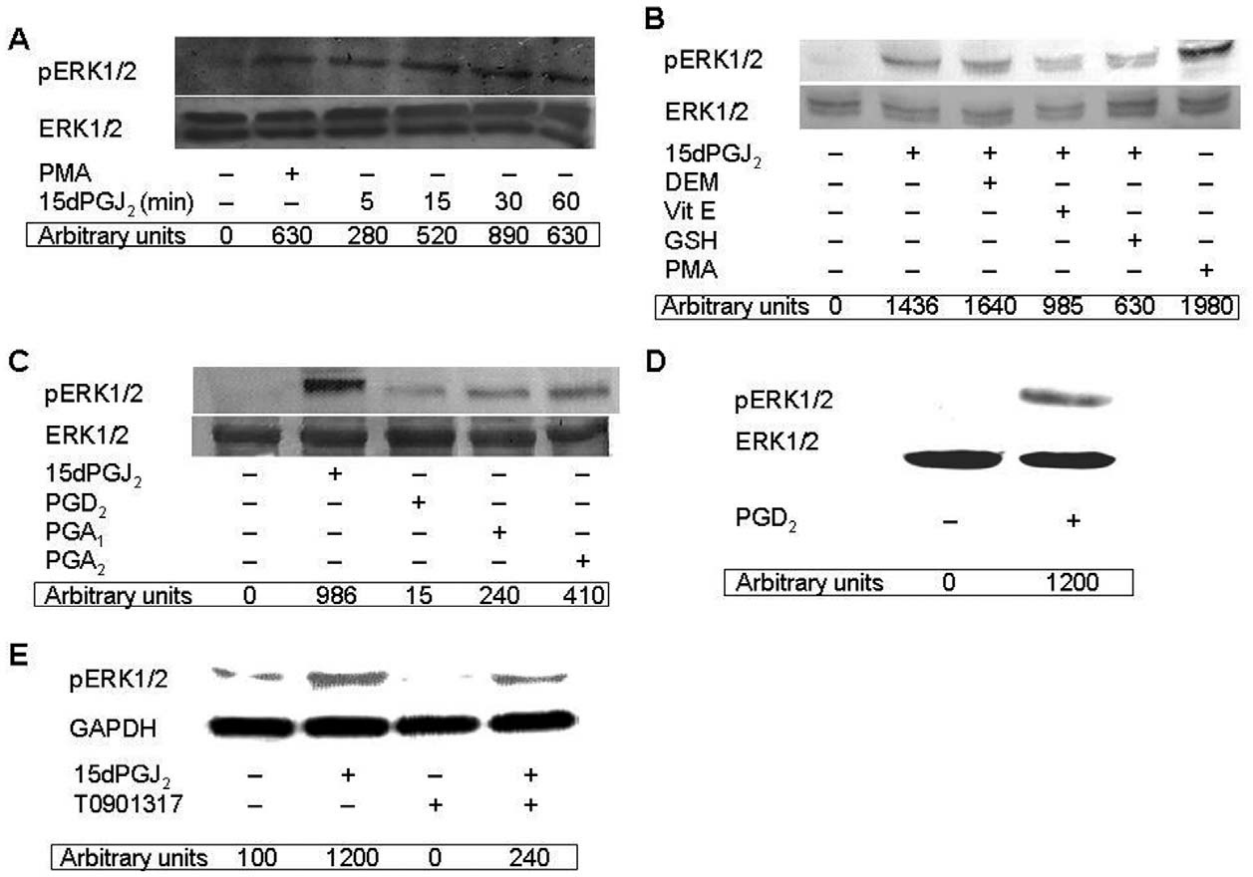
15dPGJ_2_-promoted ERK1/2 phosphorylation is dependent on oxidative stress in human neutrophils. Neutrophils were preincubated at 37°C with or without 10 µM DEM, 100 µM Vit E or 1 mM GSH for 1 h (B). Then the cells were treated or not with 10 µM 15dPGJ_2_ for the indicated times (A) or for 30 min (B, C and E), and then with 100 nM PMA for 7 min (A and B), or else with 10 µM PGD_2_, 15 µM PGA_1_ or 25 µM PGA_2_ for 30 min (C), with 10 µM PGD_2_ for 20 h (D), or with 1 µM T0901317 for 30 min (E). Phosphorylated ERK1/2 (pERK1/2) levels were analyzed on cell lysates by Western blotting and are given in arbitrary units. Immunoblots of total ERK1/2 (A-D) or GAPDH (E) are also shown for the sake of loading controls. Each blot is representative of a set of three separate experiments yielding similar results.

To further assess whether 15dPGJ_2_ was able to activate the ERK1/2 pathway in human neutrophils, we studied its effect on the phosphorylation status of this MAPK. With this purpose, neutrophils were incubated with PMA, a known ERK1/2 activator used as a positive control, and with 15dPGJ_2_ for different times (5–60 min). As illustrated in Figure 6A, 15dPGJ_2_ elicited ERK1/2 phosphorylation in a time-dependent manner, reaching its maximal value after 30 min of treatment. Given that exogenous oxidants are known to promote phosphorylation, and hence activation, of p38 MAPK and ERK1/2 in human neutrophils [48], [49], we set to analyze whether oxidative stress was implicated in ERK1/2 phosphorylation induced by 15dPGJ_2_. Thus, neutrophils were treated either with the oxidant reagent DEM, or with the reducing molecules Vit E (analog) or GSH, prior to addition of 15dPGJ_2_ to cells. As shown in Figure 6B, DEM preincubation slightly enhanced ERK1/2 phosphorylation elicited by 15dPGJ_2_, mainly of its lower, p42 band. This result was in agreement with the increased ERK1/2 phosphorylation observed upon glutathione depletion with buthionine sulfoximine in HepG2 hepatocytes [50]. On the other hand, both the Vit E analog and GSH separately hindered the positive effect of 15dPGJ_2_ on ERK1/2 phosphorylation in human neutrophils (Figure 6B). However, neither Vit E or GSH had any effect by itself on this process (data not shown). Like 15dPGJ_2_, other cyclopentenone prostaglandins, such as PGA_1_ and PGA_2_ and the 15dPGJ_2_ precursor, PGD_2_, promoted as well ERK1/2 phosphorylation in human neutrophils, although not at the high level elicited by 15dPGJ_2_ (Figure 6C). These results confirmed the possibility that 15dPGJ_2_ could act through mechanisms related to the reactive α,β-unsaturated carbonyl group in its cyclopentenone ring (Figure 6C). The much lower effect of PGD_2_ compared to 15dPGJ_2_ (Figure 6C) could be attributed to the short preincubation period (30 min) with the cells, possibly insufficient for its intracellular conversion into 15dPGJ_2_. In this light, longer preincubations, of up to 20 h, resulted in a much stronger enhancement of ERK1/2 phosphorylation by exogenously-added PGD_2_ (Figure 6D). In contrast, T0901317 was unable to stimulate ERK1/2 phosphorylation by itself (Figure 6E), but instead inhibited PMA-induced phosphorylation of this MAPK (data not shown).

### 15dPGJ_2_ Promotes LXRα Serine Phosphorylation in Human Neutrophils

**Figure 7:**
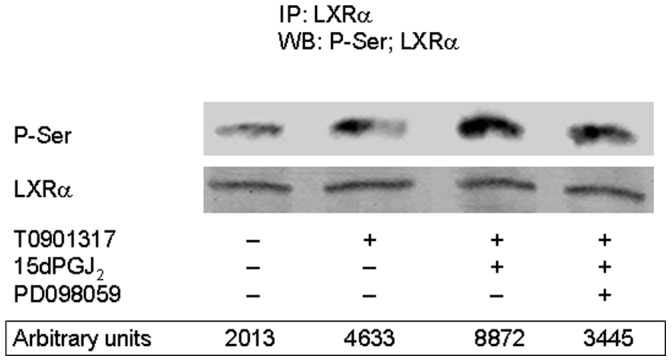
15dPGJ_2_ promotes LXRα serine phosphorylation in human neutrophils. Neutrophils were preincubated at 37°C with or without 30 µM PD098059 for 30 min. Then the cells were treated or not with 10 µM 15dPGJ_2_ for 30 min, and thereafter stimulated or not with 1 µM T0901317 for further 30 min. Finally, the cells were lysed, LXRα was immunoprecipitated and Western blotting analysis of phosphoserine (P-Ser) and LXRα levels was carried out as indicated in Experimental Procedures. P-Ser levels are given in arbitrary units. The blot is representative of a set of three experiments yielding similar results.

The human LXRα is well known to be an autoinducible gene [1], [2]. Also, nuclear receptor activity can be regulated by phosphorylation [51], [52], and previous studies have suggested that different signalling pathways may contribute to LXRα phosphorylation [53], [54]. In this context, Garabedian’s group has provided clear evidence of regulation of LXRα target gene selectivity through modulation of LXRα phosphorylation at Ser-198 in mouse macrophages [47]. Based on these data, we studied LXRα phosphorylation levels in human neutrophils. To this end, these cells were incubated with 15dPGJ_2_ prior to their treatment with T0901317, the LXRα was immunoprecipitated, and Western blotting analysis was carried out using anti -phosphorylated-serine antibodies. Figure 7 illustrates that untreated human neutrophils exhibited basal levels of LXRα serine phosphorylation, and that T0901317 incubation enhanced the serine phosphorylation status of this transcription factor. We also found that 15dPGJ_2_ further enhanced LXRα phosphorylation in human neutrophils, although at undetermined serine residue(s). It is noteworthy that this effect was counteracted by preincubation with PD098059, a ERK1/2 inhibitor (Figure 7).

## Discussion

The main role traditionally assigned to LXRs is triggering transcriptional programs that promote reverse cholesterol transport. However, as indicated above, there has been a growing appreciation in recent years of its complex roles in innate immunity using established cell lines [8], [9]. In the present work we extend the sphere of influence of LXR to human circulating neutrophils. Our studies show that when this cell type is triggered with an LXRα synthetic ligand, it exhibits a notable capacity to activate the transcription of the LXRα target genes ABCA1, ABCG1 and SREBP1c. Moreover, this ligand also induced LXRα expression itself at the mRNA and protein levels. These results confirm recent observations that identify neutrophils as target cells for LXR activation in mice [55]. These studies have demonstrated that the expression of LXRα (but not LXRβ) is controlled by an autoregulatory mechanism. Therefore, LXR activity is modulated not only by agonist and antagonist agents, but also through activation of LXR mRNA transcription exerted by LXRα itself [56]. Although most studies on LXR have been devoted to macrophages and endothelial cells, human neutrophils may play an important role in the pathogenesis of atherosclerosis, given their capacity to respond to exogenous oxysterols and circulating molecules, especially under pro-inflammatory conditions. The traditional notion of atherosclerosis as a predominantly lipid-retentive disease has been recently expanded to a coupling of inflammatory mechanisms and dyslipidemia [57]. Under this view, oxidative stress and chronic inflammation are likely involved in, and contribute to, the development of atherosclerosis [58], [59]. In this context, primed neutrophils constitute a source of superoxide radicals (O_2_. ^−^) and mediators that can promote oxidative stress and inflammation [34], whose overproduction may represent a risk factor for cardiovascular diseases [60]. The LXR nuclear receptor is generally considered as an anti-inflammatory factor, given that it hinders the expression of classical pro-inflammatory genes [8], In this line, we show evidence that T0901317 treatment was able to ameliorate neutrophil migration activity induced by pro-inflammatory agents (such as 15dPGJ_2_ and fMLP). Moreover, T0901317 treatment reverted the pro-inflammatory effect of 15dPGJ_2_ (i.e. ROS production, IL-8 release), But it is important to underline that these effects occurred only after long term treatment with T0901317 (i.e. 4 h). Thus the activation of LXR may play a role in resolution of inflammation processes.

Although it has been shown that under oxidative stress conditions the NADPH oxidase system becomes activated in murine macrophages treated with oxysterols [61], and that in cultured porcine retinal pigment epithelial cells oxysterols induce a significant increase of ROS production and IL-8 gene expression [62], the discrepancy existing on the relationship between oxysterols and oxidative stress could reflect differences between animal species or cell lines in their response to these compounds. However, the opposite situation, that is, the potential effect of oxidative stress on LXR gene expression, has not been analyzed in human circulating neutrophils and constitutes one of the aims of the present study. The main role of neutrophils as a source of ROS (e.g., superoxide radicals) and pro-inflammatory mediators (e.g., 15dPGJ_2_), thereby promoting oxidative stress and contributing to the development of atherosclerosis, is well known [34]. Therefore, these cells offer a natural approach to analyze the potential effect of ROS on LXR transcription in human cells, by using 15dPGJ_2_ as a pro-inflammatory molecule. As indicated, 15dPGJ_2_ could be considered either as a pro- or anti-inflammatory molecule depending on cells under study, their context and the prostaglandin receptor implicated [63]. However, our present data provide clear evidence that 15dPGJ_2_ exerts pro-inflammatory effects in human neutrophils, since it induces ROS production at high levels by this cell type. This effect was dependent on its cyclopentenone ring, and in this context it has been reported that some 15dPGJ_2_ biological actions involve the covalent modification or oxidation of critical cysteine residues acting as a redox sensor [64]. Additionally, 15dPGJ_2_ is able to activate cytoprotective mechanisms, such as the induction of HO-1 expression, in an NF-κB-independent fashion [30]. Similar results have been described by our group in human lymphocytes [32], and by other researchers in murine macrophages [65]. Therefore, accumulating evidence of 15dPGJ_2_ pro-inflammatory actions [22], [23], [66], [67] taken altogether makes its anti-inflammatory properties increasingly controversial [24]–[27], [31].

Two main observations were made in the present study regarding regulation of LXRα gene expression in human circulating neutrophils. First, these cells were able to respond positively to T0901317 by up-regulating the mRNA levels of LXRα target genes by several-fold together with its transcriptional transactivation activity. Second, we describe for the first time that LXRα mRNA transcription induced by its ligand is inhibited by 15dPGJ_2_ in a fashion similar to other model pro-inflammatory molecules, such as IL-8, TNFα and IL-1, which inhibit LXR transcription in human neutrophils, as similar manner as its do on Hep3B human hepatoma cells [68]. With Regard to potential mechanisms whereby 15dPGJ_2_ acts negatively on LXRα target genes mRNA expression, present data suggest that its inhibitory effect is exerted in a PPARγ-independent manner, because, other PPARγ ligands do not have any effect on LXRα expression. These data are in agreement with the fact that anti-atherogenic effect of PPARγ ligands are exerted in a LXR-independent manner in macrophage foam-cell formation [69]. Furthermore, our data also suggest that 15dPGJ_2_ inhibitory effect is performed in a ROS-dependent manner. Several lines of research illustrate the possibility that 15dPGJ_2_ effect could be exerted in a PPARγ-independent and ROS-dependent manner [70], [71]. We have found that such 15dPGJ_2_ negative effect was reversed by reducing agents and mimicked by pro-oxidant molecules, this reflecting a previously undescribed relationship between ROS production and modulation of LXR expression. This observation could be very important in the context of pathologies associated involving an inflammatory process, since the relationship between LXR activation and atherogenesis counteraction associated with reverse cholesterol transport stimulation by LXRs is well known [56], [72]. In addition, it is also noticeable the relationship between ROS production and atherosclerotic vascular impairment [58], [59], [73]. Therefore, we can postulate that ROS production by neutrophils occurring in different inflammatory processes might interfere with other LXR-regulated events, such as atherosclerosis enhancement or even its anti-inflammatory effect. This is evidenced by the fact that inhibition of LXRα, by increased pro-oxidant condition, reversed the LXRα anti-inflammatory effects on IL8 release and ROS production induced by 15dPGJ_2_. In fact, this idea has already been raised by other researchers concluding that ROS may promote coronary artery disease by counteracting the established anti-atherogenic effects of HDL and ABCA1 pathways on the human artery wall [74]. Our data are also consistent with recent reports that ROS mediate the IL-1β-induced down-regulation of ABCA1 mRNA and protein in human macrophages (THP-1 and A549 cell lines) [75], and that epigallocatechin-3-gallate, which elevates ROS in the 3T3L1 adipocyte cell line, also decreases LXRα expression [76]. However, the latter study did not address whether this inhibitory effect was or not ROS-dependent.

15dPGJ_2_ did not negatively modulate only LXRα expression, but also hindered mRNA transcription of its target genes ABCA1, ABCG1 and SREBP1c in T0901317-stimulated human neutrophils (although its effect on SREBP1c was not statistically significant). These data are in agreement with the well known fact that these three genes are LXR-regulated in rodent and human cells, via functional LXR response elements found in their promoters [7], [56], [77]. Therefore, it seems that 15dPGJ_2_, by negatively modulating LXRα transcription factor activity, disables the expression of LXR target genes. Additionally, LXR activity appears to be regulated by post-translational modification, such as phosphorylation exerted by MAPKs, as it is the case for other nuclear receptors [51], [78], [79]. This led us to analyze the potential implication of these protein kinases in the modulation of LXR mRNA expression by testing the effect on this process of a set of MAPK inhibitors targeting p38, ERK1/2 and JNK1/2. Among these, neither SB203580 nor SP600125 had any effect on LXRα transcription, which reflected the lack of implication of p38 MAPK and JNK1/2. However, we found that treatment of neutrophils with PD098059, an inhibitor of ERK1/2 upstream activator, MEK1/2, resulted in a dramatic enhancement of mRNA synthesis of LXRα and other target genes induced by T0901317. Even, PD098059 alone was able to slightly increase transcription of these genes. In contrast, it has been very recently described in macrophages that ERK1/2 inhibitors do not affect LXRα/β protein expression, although they increase free cholesterol efflux and ABCA1 expression in this cell type [80]. Additionally, we present evidence that PD098059 potently cancels 15dPGJ_2_ inhibition of mRNA synthesis of LXRα and other target genes. These data strongly suggested that ERK1/2 activation is involved in the down-regulation of T0901317-induced transcription LXRα of target genes exerted by 15dPGJ_2_ in human neutrophils. In fact, we have detected that 15dPGJ_2_ elicited ERK1/2 phosphorylation in a PPARγ-independent manner, in similarity to reports in other cells [81], [82], and that this effect was dependent on ROS production. This conclusion was further supported by the fact that other down-regulator of LXRα target genes used in this work, such as other prostaglandins, promoted as well ERK1/2 phosphorylation in human neutrophils. Therefore, in these cells LXR activity appears to be regulated, in addition to at the transcriptional level, through post-translational modification elicited by T0901317 and 15dPGJ_2_. In this context, we have found that the LXRα protein undergoes serine phosphorylation promoted by its ligand, T0901317. This result is in line with the previous observation that selective regulation of LXRα target gene expression, rather than expression of the LXRα gene itself, is achieved in macrophages by phosphorylation of the LXRα at Ser-198 [47], a residue located within a consensus target for the MAPK family [46]. Our data in human neutrophils indicate that 15dPGJ_2_ treatment induces an increased phosphorylation at undetermined serine residue(s) of the LXRα.

In summary, our results support the notion that pro-inflammatory stimuli such as 15dPGJ_2_, together with associated ROS production, down-regulate the LXRα and at least some of its other target genes at the mRNA expression level in human neutrophils by promoting an alteration of the LXRα phosphorylation status in an ERK1/2-dependent manner. Whichever the action exerted by oxysterols (i.e., pro- versus anti-inflammatory), experiments in this work clearly indicate that LXR transcription becomes depressed under oxidative conditions. This is highly suggestive that neutrophils, alike other phagocytic cells, could be associated with the high oxidative state found in atherogenic injury conditions [58], [59], [73]. This pro-oxidant environment would promote the formation of the atherosclerotic plaque through the direct negative modulation of LXR activity. Present studies we believe open up the new paradigm of a cross-talk between nutritional transcription factors modulating fatty acid metabolism and oxidative stress in human circulating blood cells.
